# Structural-Scaling Transitions and Criticality Cascade in DNA with Open States

**DOI:** 10.3390/ijms26178428

**Published:** 2025-08-29

**Authors:** Aleksandr S. Nikitiuk, Yuriy V. Bayandin, Oleg B. Naimark

**Affiliations:** 1 Laboratory of Physical Foundations of Strength, Institute of Continuous Mechanics UrB RAS, Perm 614013, Russia; buv@icmm.ru; 2 Applied Mathematics and Mechanics Faculty, Perm National Research Polytechnic University, Perm 614990, Russia

**Keywords:** DNA, open state, gene expression, statistical thermodynamics, mechanobiological model, self-organized criticality

## Abstract

This article investigates the mechanism of self-organized DNA criticality with open states, which plays a key role in the regulation of gene expression and consequently in cell fate determination. Based on a mechanobiological model developed using methods of statistical physics and thermodynamics, we demonstrate that the collective behavior of DNA open-state ensembles governs transitions between bistable, metastable, and critical genomic states. These states correspond to different gene expression scenarios involved in cell fate determination. Through simulation results, we introduce the concept of a criticality cascade, linking the dynamics of the DNA molecule structural parameter χ with global changes in cellular processes. The findings align with experimental data and offer new perspectives for studying genome regulation mechanisms, including pathological conditions such as cancer.

## 1. Introduction

Understanding the fundamental principles of cell state transitions during development and differentiation processes is a fundamental issue in biology involving basic principles of the physics of living systems. Beyond its fundamental biological relevance, the societal and economic importance of DNA technologies is also rapidly increasing. According to Grand View Research, the global genomics market was estimated at USD 35–45 billion in 2024 and is projected to reach USD 80–120 billion by 2030. This growth is driven by advances in healthcare, agriculture, and forensic applications, thereby contributing to improved disease management and food security.

The process that results in a stable cellular state can be modeled as a regulated molecular cascade that ensures the reaching of the proper biological function of the cell like in the differentiation process. Other molecular cascades foster the onset of proliferation, *G*_0_ phase arrest (dormancy), senescence, or apoptosis.

Beside the huge differences in the physiological meaning of these processes, we can individuate three fundamental common stages: initiation under the influence of external or internal signals, activation of signaling pathways, and a change in cell fate (e.g., transitioning from a state of differentiation to proliferation). At the same time, these cellular processes depend on the coordinated expression of thousands of genes, whose collective organization principles are largely independent from the involvement of particular gene products. Unlike inanimate objects that undergo phase transitions, no universally accepted physical model connects the system’s energy to gene expression modulation as of yet. However, phenomenological models, which involve drastic changes in entropy and system correlations, provide consistent results [1,2].

These phenomenological models build on the idea of self-organized expression of thousands of genes. This concept was first proposed in [3]. In [4], the authors laid the foundations for gene regulatory networks (GRNs), where coordinated gene expression arises from interactions between regulatory elements. With the advancement of sequencing technology, the idea of global self-organization of the transcriptome has received experimental confirmation [5,6]. Huang et al. [7] proposed the concept of attractors in GRNs, using it to link stable cell states to the epigenetic landscape determined by gene expression. An enhanced comprehension of DNA self-assembly and motif stability [8] will facilitate the development of DNA-based drug delivery platforms [9]. In these platforms, controlled open states could enable targeted release mechanisms, thereby enhancing therapeutic efficacy.

A series of studies [10,11,12,13,14,15] used the concept of self-organized criticality to explore gene expression regulation. They established a general mechanism for coordinated regulation at the whole-genome level. This mechanism leads to cell fate changes and was observed in embryonic, immune, and cancer cell populations, as well as isolated cultured cells. Goldenfeld and Woese introduced a nonequilibrium statistical physics framework [16,17,18]. Building on this, recent studies show that a small ensemble of ‘critical point’ genes acts like a Maxwell’s demon—a rewritable chromatin memory that synchronizes genome-wide expression during cancer cell fate transitions [19]. However, the detailed biophysical mechanisms, particularly how chromatin remodeling complexes implement this entropy-driven information flow, remain to be fully elucidated.

Some authors [20,21,22,23,24,25] have proposed theoretical approaches to this issue. They suggest that the mechano-biology of DNA serves as the physical basis for collective gene expression regulation. This regulation stems from the mutual influence of DNA open-state ensembles. These studies propose a physical mechanism for deterministic DNA behavior and identify collective open-state modes that support DNA function. Our study applies a mechanobiological model to explore thermodynamic regulation of whole-genome expression. The model is nonlinear and adopted the framework of statistical physics and out-of-equilibrium thermodynamics. The results presented in this study provide a reliable physical basis for the mechanism of self-organized regulation of whole-genome expression for a wide range of cell types, as proposed in a series of studies [10,11,12,13,14,15]. They also allow for the prediction of cell fate changes during transitions between multiple cellular states.

The paper is structured as follows. Section 2.1 explores the experimentally validated mechanism of self-organized regulation of whole-genome expression. It draws on gene expression dynamics observed in embryonic, immune, and cancer cell populations, as well as isolated cultured cells. This section introduces the concept of critical-point genes acting as a Maxwell’s demon-like mechanism to synchronize genome-wide expression. Section 2.2 defines DNA open states and their internal variables, such as base-pair displacement and supercoiling stress. It presents the general formulation of our mechanobiological model, which quantifies open-state dynamics using thermodynamic principles.

Section 2.3 examines the collective modes of open states, describing how they drive self-organization through structure–scaling transitions. These transitions reflect changes in DNA’s structural parameter χ, influencing genome-wide dynamics [20]. Section 2.4 presents numerical simulations of self-organized DNA criticality, modeling a full-length DNA molecule (approximately 3 billion base pairs) to demonstrate qualitative scenarios of state transitions. Section 2.5 provides a thermodynamic analysis of this criticality, detailing how entropy and energy shifts govern open-state behavior. Section 2.6 introduces the criticality cascade, a novel effect predicted by our model, where local structural changes trigger global genomic shifts. This section clarifies that simulations use a single deterministic run to highlight bifurcation points and self-similar solutions, as described in Appendix A.

Section 2.7 proposes experimental approaches to validate the model, such as magnetic tweezers to measure open–closed-state transitions and fluorescence microscopy to monitor real-time DNA dynamics. These align with existing data and complement the theoretical framework. The Discussion section summarizes key findings, their implications for genome regulation, and future research directions, including potential applications in cancer studies. The Conclusion reflects on the study’s contributions and its broader impact on understanding cellular processes.

## 2. Results

### 2.1. Genomic Mechanism Determining Cell Fate

Using the results reported in [14] as an example, let us examine the experimentally established mechanism of gene expression regulation at the whole-genome level, which determines changes in cell fate. The analyzed case studies, demonstrating the universality of the discovered principle, include single cell and cell population data coming from human and mouse embryos, immune (Th17), and cancer (MCF-7, HL-60) cell types. The authors [14] demonstrated how a self-organized criticality (SOC) phenomenological model allows for a thorough description of whole genome expression regulation, accounting for large-scale coordinated changes in mRNA expression levels of thousands of genes. Additionally, universal principles underlying the switching of cellular states (e.g., proliferation → differentiation) were identified, and it was explained how minor external influences can trigger global changes in the genome through the cooperative dynamics of a gene group acting as an organizing center for cell fate. It is worth noting how these results are consistent with the concept of Network Dynamical Biomarkers (DNB) theory, independently developed by the group of Luonan Chen [26,27] suggesting the presence of a surge of ‘internal correlations’ within a group of system elements acting as ‘organizers’ of the global behavior of the system at hand.

The datasets analyzed in [14] correspond to the gene expression vectors measured at various time points. From these initial data, the normalized root mean square fluctuation (*nrmsf*) was calculated for each gene to characterize its fluctuation level, as well as the center of mass (mean) of the expression vector at a specific time. The *nrmsf* of a gene is a proxy of the relative flexibility of the chromatin domain it pertains, so it acts as a link between expression regulation and structural features of the genome. The genome can be conditionally divided into three gene groups on the basis of their relative *nrmsf* value as a sub-, near-, or super-critical state, identified as unimodal, flattened unimodal, or bimodal probability distribution functions of the change in the gene expression vector between two time points. The interaction between these three gene groups defines the “genome engine”, which governs cell fate.

To identify coordinated changes in gene expression, correlation analysis based on the Pearson pairwise correlation criterion was applied, and two-dimensional probability density functions for expression vectors at two different time points were constructed. Approaches from dynamical systems theory, such as expression flux analysis, were also used to study the interaction between critical states arising among three different gene groups of the whole genome (super-, near-, and sub-critical states). The application of these approaches led to the proposal of a “genome engine” mechanism that ensures autonomous regulation of genome expression.

The key results of the study can be summarized as follows. The expression of each individual gene is stochastic. However, expression changes involving thousands of genes happen in a highly coordinated way starting from a subset of ‘super-critical’ genes transmitting their expression changes to the entire genome.

At the critical point, a singular behavior starts to appear: the super-critical organizer genes oscillate between two ON/OFF states. The switching between these two states leads to an avalanche-like gene expression, which results in a change in cell fate.

These results imply that cell fate is determined not by individual gene regulation but by coordinated changes in the critical states of the genome, arising through the SOC mechanism. This explains how cells can quickly and coherently switch between different states.

Based on the obtained results, several prospects for further research can be highlighted. First, it is necessary to study the molecular mechanisms underlying the activation and deactivation of the critical point, including the role of chromatin remodeling. Second, the obtained results and the conclusions drawn from them require validation in other biological systems, such as induced pluripotent stem cells (iPS). Third, it is necessary to develop a theoretical approach explaining the genome’s ability to self-organize through transitions between critical states. The latter can be investigated using a statistical-thermodynamic approach, which adequately describes living systems as macroscopic, out-of-equilibrium, and open.

### 2.2. DNA Open States

The DNA molecule has a helical shape and is formed by a sequence of nucleotide pairs. Each nucleotide consists of three elements: a phosphate group, a sugar ring (a five-membered cyclic group), and a base, which is a complex organic group that may contain one or two rings. The phosphate group and sugar ring are connected by covalent bonds to form the sugar-phosphate backbone. Nitrogenous bases pair through hydrogen bonds according to complementary principles, forming pairs such as adenine-thymine and guanine-cytosine.

In the equilibrium configuration, the action of these bonds stabilizes and determines the quasi-symmetric helical structure of DNA. One of DNA’s primary functions is the realization of hereditary information encoded in the unique sequences of bases. To perform this function, DNA must displace a base or group of bases from their equilibrium positions, making them accessible for reading by DNA or RNA polymerase through partial or complete disruption of hydrogen bonds [28]. This act represents a local symmetry breaking in the DNA structure.

To describe the symmetry breaking properties in DNA, the displacement of a nitrogenous base from its equilibrium position in a nucleotide pair is associated with a vector **y**(*r*,φ,θ), defined in a cylindrical coordinate system. The magnitude of base displacement relative to the equilibrium position can vary: for a denaturation bubble, it ranges from 4 to 6 angstroms; for a flip-out or water-bridge complex, it involves a 45-degree rotation around the attachment point to the sugar-phosphate backbone. In the cases involving all degrees of freedom, the displacement magnitude is estimated at approximately 3 angstroms (determined by the ability to accommodate a water molecule) [28]. An open state corresponds to any displacement of a nitrogenous base in a nucleotide pair from its equilibrium position greater than 3 angstroms. This displacement is favored under conditions of mechanical stress, defined as a force greater than 10 pN, or elevated temperatures, specifically between 80 and 90 degrees Celsius, a range that is particularly relevant for AT-rich sequences. Conversely, if the base pair is found to be in a closed state, it is considered to be in a closed state.

**Figure 1:**
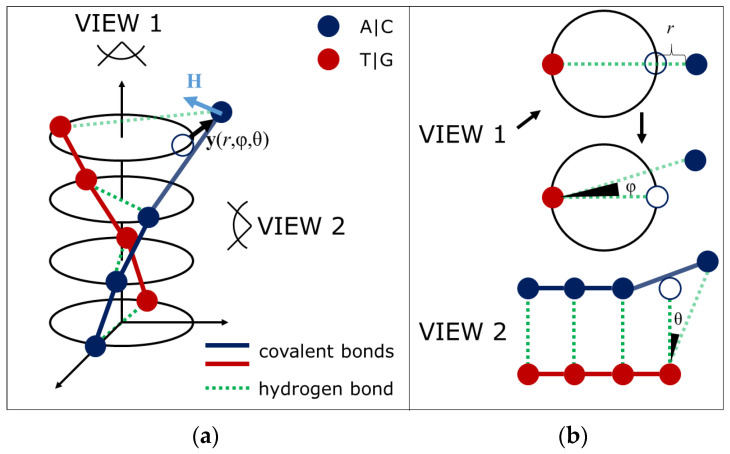
Schematic representation of DNA with an open state. (**a**) Shows a DNA segment with one open and three closed nucleotide pairs. Red/blue circles represent different bases; green dashed lines are hydrogen bonds; solid lines are covalent bonds. The open state has a displaced base. (**b**) Side view of DNA.

Figure 1 provides a schematic representation of a stretch of DNA with one open and three closed states. Red and blue circles denote nucleotides differing in the type of nitrogenous base—adenine or cytosine (red) and thymine or guanine (blue), respectively. Green dashed lines represent hydrogen bonds formed between complementary bases. Red and blue solid lines depict covalent bonds. One nucleotide pair is in an open state (upper plane), while the other three are in closed states. A closed state is characterized by the equilibrium position of the bases, specifically, for B-DNA, the distance between bases is approximately 12 angstroms, the twist angle is 36 degrees, and the distance between nucleotide planes is 3.4 angstroms. Any displacement of a nucleotide base exceeding 3 angstroms in absolute value is considered an open state (Figure 1b).

The formation of open or closed states in DNA creates an internal field **H**, which acts on other regions of the molecule and promotes conformational changes (Figure 1a). Three physical mechanisms can explain the emergence and action of this field. The first is thermodynamically driven cooperativity of “helix-coil” transitions. Complete closure of DNA reduces the system’s entropy, increasing the likelihood of the remaining open states closing. Conversely, complete opening increases the system’s entropy, facilitating the opening of the last closed states. In the study [29] on DNA denaturation (melting), it was established that transitions between open and closed states depend on the degree of openness/closure of neighboring regions. Research [30] on stretching native DNA confirms that the opening of one DNA region facilitates the opening of adjacent ones. The second mechanism arises from electrostatic interactions between neighboring nucleotide pairs in closed states and the charged phosphate groups that become accessible upon the formation of open states [31], due to increased electrostatic tension [32].

The third mechanism involves the influence of mechanical stress caused by supercoiling and bending of the molecule [33], which propagates along the chain [34,35]. These effects explain how changes in one part of DNA can influence other regions through an internal field.

### 2.3. Collective Properties of the Open States Ensembles

The statistical description enabled the proposal of a DNA phenomenology with open states, based on the free energy representation Ψ. In consideration of the results presented in the preceding section, the mathematical formalism is reported in Appendix A.

Here it is sufficient to stress that the mathematical formalism implies the presence of two bifurcation points, namely χ*_t_* and χ*_c_* that play a role analogous to characteristic temperatures in Landau’s theory of phase transitions and mark abrupt changes in the system structure.

Thus, these results give an explanation of how the collective behavior of open states, governed by the structural parameter χ, drives the system through different critical regimes. The self-similar solutions represent the finite amplitude fluctuation of the open-state order parameter in different areas of metastability (criticality), which provide different scenarios of Maxwell’s Demon-Like Regulation of Cell-Fate Transition [19]. It is worth reminding that the notion of ‘Maxwell’s Demon’ comes from a thought experiment introduced by James Clerk Maxwell [36]. The demon (acting as an ‘intelligent agent’) discriminates ‘hot’ and ‘cold’ molecules in a recipient allowing only one type of molecule to transit a barrier subdividing the recipient into two chambers. This produces a ‘hot’ and a ‘cold’ chamber thereby reducing entropy and violating the second law of thermodynamics. In crowded populations, such as in vivo chromatin, collective open states are amplified by macromolecular crowding, reducing entropy and promoting cooperativity, unlike isolated single strands where modes are less correlated.

Toyabe et al. [37] first experimentally demonstrated the conversion of information into usable work via real-time feedback control, effectively realizing a modern Maxwell’s demon using a colloidal particle system.

Far from being a mere metaphor, Maxwell’s demon embodies a functional principle, underscoring the profound connection between information processing and thermodynamic behavior in non-equilibrium systems, including biological phenomena. It is worth noting that in a largely unconscious way, the majority of biological research papers involve the action of ‘Maxwell Demons’ like agents like transcription factors that ‘recognize’ specific DNA sequence tracts or protein molecules that ‘recruit’ other protein species in order to build up molecular aggregates performing a specific task. This is why the ‘Intelligence’ metaphor can be of use to describe biological phenomena [38]. Here we are able to ‘give-a-name’ to such ‘material intelligence’ in terms of statistical physics.

### 2.4. Numerical Simulation of the Self-Organized DNA Criticality

In order to simulate the dynamics of DNA with open states, the entire genome was divided into three interacting representative segments, with the dynamics of open states in each segment determined by the system of equations (B20), but without accounting for chemical influence (the last term on the right-hand side of the second equation in the system).

**Figure 2:**
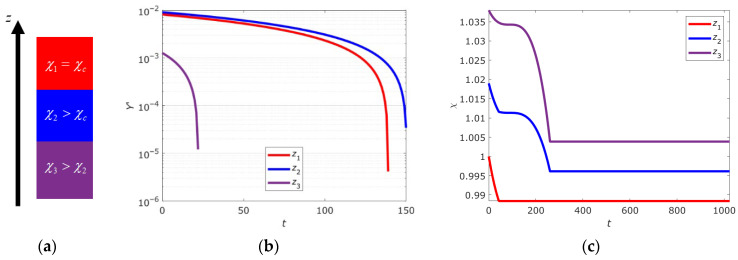
Schematic representation of the genome divided into three representative sites (**a**) and dynamics of changes in Y’ (**b**) and χ (**c**) for each of the selected sites.

The dynamics of the dimensionless macroscopic parameter *Y*’ and the structural parameter χ for the three DNA segments are illustrated in Figure 2. The segment labeled *z*_1_ corresponds to a bistable state (χ_1_(*t*_0_) = 1.038), *z*_2_ to a metastable state (χ_1_(*t*_0_) = 1.019), and *z*_3_ to a supercritical state (χ_1_(*t*_0_) = 1) (see Figure 2a). The graphs depict the evolution of open-state ensembles for different DNA segments. Both model parameters decrease over time for all segments. The decrease in *Y*’ values (Figure 2b) is associated with the system transitioning to a state with a lower degree of openness, driven by the chosen initial conditions. In all three cases, the subsystems are in near-stable states, corresponding to the free energy minimum near zero on the abscissa axis. As a result, a cooperative effect of closing open states occurs under the influence of the internal field **H** across the entire genome. As noted in Section 2.2, this behavior aligns with the thermodynamically driven cooperativity of transitions, where the closure of one DNA segment reduces the system’s entropy and promotes the closure of others.

Thus, the presented results are consistent with the theoretical predictions outlined earlier and confirm the role of the structural parameter χ as a key factor determining the type of collective behavior of open states. In the absence of a chemical signal, this parameter can only degrade over time (see Figure 2c), as dictated by the evolutionary Equation (21)_2_. The plateau in the structural parameter values is due to reaching the critical value χ*_c_*. Due to discretization, numerical effects, and boundary conditions, the plateau levels differ between DNA segments. For instance, finite-difference approximation “smears” sharp transitions over several grid nodes; the fifth-order implicit Gear method introduces additional numerical error; and second-kind boundary conditions create a boundary layer that modifies local behavior. This means the critical value χ*_c_* = 1 corresponds to an idealized case of an isolated and single representative DNA segment.

### 2.5. Bistable, Metastable and Critical States of DNA

**Figure 3:**
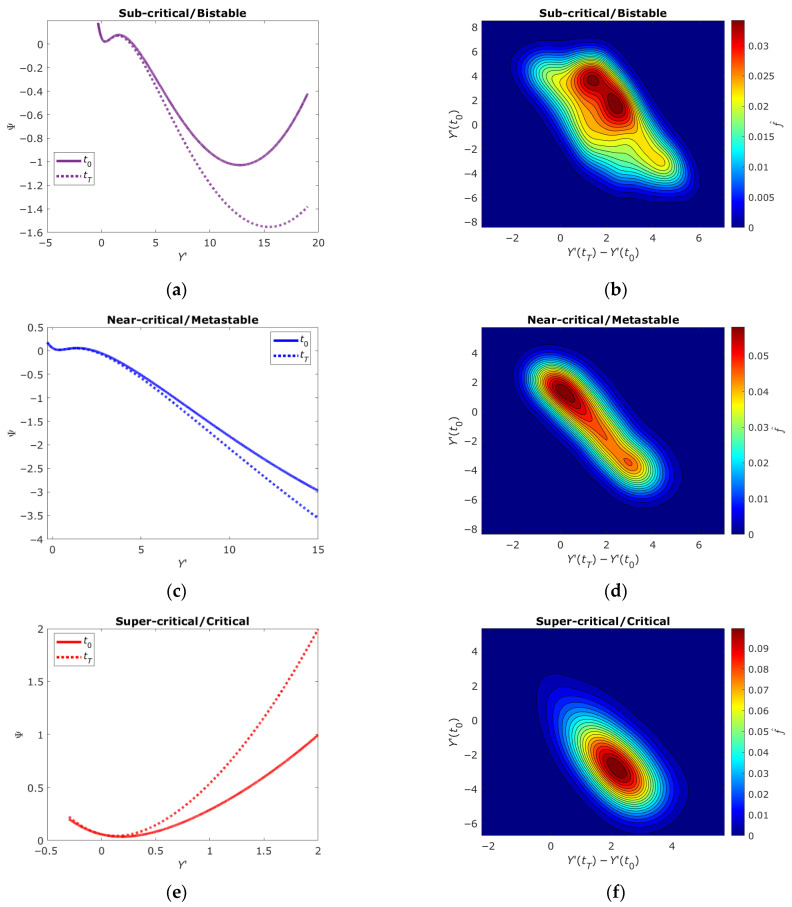
Profiles of the thermodynamic potential (**a**,**c**,**e**) and their corresponding probability density functions (**b**,**d**,**f**) for the cases of bistable, metastable and critical states of DNA.

Section 2.4 presented numerical simulation results of open-state ensemble behavior in three representative DNA segments with different initial values of the structural parameter χ. These values determine three characteristic system functioning regimes: bistable, metastable and critical, corresponding to the sub-, near- and super-critical gene expression domains previously identified in works [10,11,12,13,14,15]. These regimes reflect different types of collective behavior of open-state ensembles [20,21,23,24,39]. Based on the calculated dependencies *Y*’(*t*) and χ(*t*) shown in Figure 2, using the free energy Equation (10) we can construct thermodynamic potential profiles Ψ(*t*), with calculation results shown in Figure 3a,c,e.

If the form where the thermodynamic potential is known, it becomes possible to estimate the statistical properties of the system as a whole and its individual components. This allows qualitative comparison with experimentally established gene expression patterns, linking the proposed model representations with specific measurable quantities and revealing their physical nature. As noted in Section 2.1, the genome can be conditionally divided into three gene groups, each in sub-, near- or super-critical states identified by unimodal, flattened unimodal or bimodal probability distribution functions of gene expression vector changes between two time points. Accordingly, the probability distributions of changes in internal thermodynamic variables of DNA open-state ensembles *Y*’, whose evolution is determined by one of the thermodynamic potential types (Figure 3a,c,e), will correspond to the probability density functions identified in gene expression analysis.

Comparing probability density functions of gene expression vector changes and internal thermodynamic variable vectors of DNA open-state ensembles between two time points is justified because gene expression depends on DNA region accessibility and transcription kinetics, including initiation rate, elongation and mRNA degradation. This suggests that mRNA synthesis frequency measured in experiments [10,11,12,13,14,15] is proportional to the number or average size of open states in a gene in the absence of complex regulatory mechanisms.

The algorithm for constructing probability density functions of changes in internal thermodynamic variable vectors of DNA open-state ensembles Y’ for three different thermodynamic potentials (Figure 3a,c,e) includes the following steps. First, the form of the normalized partition function *Z*(*Y*’) is determined through the relation Ψ(*Y*’)~−ln(*Z*(*Y*’)) as:

(1) $$Z\left(Y^{\prime }\right)≃\frac{\exp\left(-\Psi \left(Y^{\prime }\right)\right)}{\int \exp\left(-\Psi \left(Y^{\prime }\right)\right)dY^{\prime }}$$

Then using the *Z*(*Y*’) distribution, random values of variable *Y*’ are generated, which may correspond to DNA segments equal in length to one gene. This procedure is applied to obtain vectors of internal thermodynamic variables of DNA open-state ensembles at initial and final time points. Based on this data, the probability density function of *Y*’ with a Gaussian kernel is calculated according to

(2) $$\hat{f}\left(Y\prime_{x},Y\prime_{y}\right)=\frac{1}{K}\sum_{i=1}^{K}\frac{1}{2\pi h}\exp\left(-\frac{{\left(Y\prime_{x}-Y\prime^{i}\left(t_{j}\right)\right)}^{2}+{\left(Y\prime_{y}-Y\prime^{i}\left(t_{j+1}\right)\right)}^{2}}{2h^{2}}\right),$$

where *K* is the number of DNA segments, *Y*’*_x_* and *Y*’*_y_* are grid values where the probability density function will be calculated, *h* is the smoothing parameter, *i* is the DNA segment (gene) number, *t* is the time, and *j* is the time point number (initial time point corresponds to *j* = 0, final time to *j* = *T*).

Results of open-state probability density function calculations are presented in Figure 3b,d,f showing probability density functions corresponding to each thermodynamic potential profile Ψ. The functions are constructed for *Y*’ at initial time and differences in *Y*’ values between final and initial simulation time points. The smoothing parameter *h* was set to 0.7. The number of DNA segments *K* for which random values characterizing their openness degree were generated according to free energy profiles was 512.

The bistable state (χ_0_ = 1.038) is characterized by two stable minima in the free energy profile (Figure 3a), corresponding to two possible DNA conformational states: predominantly closed or open. This behavior agrees with the sub-critical regime where the system can reside in one of two stable states separated by an energy barrier. The probability density function (Figure 3b) demonstrates a bimodal distribution, confirming possible switching between these states. This indicates that in the bistable regime DNA is capable of cooperative transitions that may play a key role in gene expression regulation.

The metastable state (χ_0_ = 1.019) appears as a free energy profile with one stable and one unstable minimum (Figure 3c). This regime corresponds to near-critical behavior where the system demonstrates intermediate properties between bistability and criticality. The probability density function (Figure 3d) has a flattened unimodal distribution, indicating increased system sensitivity to external influences. In this state, DNA can easily switch between different conformations under the influence of internal field **H** or external signals, which is important for dynamic regulation of genomic processes.

The critical state (χ_0_ = 1) is characterized by a super-critical free energy profile (Figure 3e) lacking local minima, with the system near a phase transition. The probability density function (Figure 3f) has a pronounced unimodal distribution, indicating absence of stable conformational states. In this regime, DNA demonstrates maximum sensitivity to small perturbations, which may lead to avalanche-like changes in gene expression and global cellular state rearrangements.

Analysis of statistical distributions confirms that gene expression is determined by collective behavior of open-state ensembles, independent of temperature but regulated by the structural parameter [20,21,23,24,39]. Bistable, metastable and critical states correspond to different genome regulation regimes. Transitions between these states may be caused by χ changes, consistent with self-organized criticality concept. Internal field **H** plays a key role in cooperative closing or opening of DNA regions, confirmed by decreasing *Y*’ and χ values over time (Figure 2). The results qualitatively agree with experimental data describing mechanisms of cellular state selection through gene expression changes. For example, the bistable state may correspond to switching between proliferation and differentiation, while the critical state corresponds to global genome changes. Thus, the proposed model allows describing different DNA behavior regimes and explaining how collective interactions of open states may influence genome regulation and cell fate changes.

The thermodynamic profiles of DNA states (Figure 3) gain deeper significance when contextualized with recent work on genomic entropy flows. Tsuchiya et al. [19] showed that CP genes act as entropy reservoirs, synchronizing with transcriptome-wide phase transitions—a process mirrored in our model’s χ-dependent criticality cascade. Similar results were obtained in [40] at the single cell level giving further confirmation to our model. Furthermore, the study [41] demonstrated that chromatin structural transitions mediate this synchronization, aligning with our bistable/metastable classifications. Together, these studies suggest that DNA’s mechanical criticality (governed by χ) and thermodynamic criticality (governed by entropy/information exchange) are intertwined facets of genome regulation.

### 2.6. Criticality Cascade in DNA with Open States

As noted in Section 2.1, global gene expression changes are regulated by a specific gene group whose dynamics in phase space forms an attractor set determining the behavior of the entire genomic system. This group can be identified as a critical point corresponding to the maximum of the two-dimensional gene expression probability density function. According to Section 2.5 results, this point can also be characterized through the thermodynamic potential minimum, allowing analysis of global minimum *Y*’_min_(t) dynamics of the Ψ_global_ function. This function can be defined by summing potentials of all representative DNA segments, due to the free energy additivity property.

**Figure 4:**
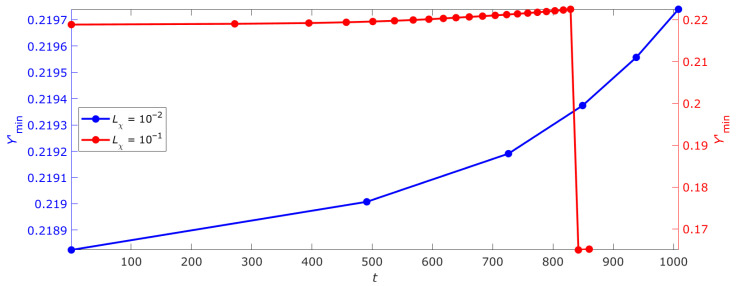
Time dependence of the global minimum of the thermodynamic potential of DNA.

Simulations model the full-length DNA as an ensemble of open states, with results from a single deterministic run to demonstrate qualitative criticality scenarios. Figure 4 shows the dependence of global thermodynamic potential Ψ_global_ minimum on time, demonstrating nonlinear growth of the *Y*’_min_ value; no statistical deviations are applicable due to the non-stochastic nature (see Appendix A for equations). This dynamic reflects critical point movement determined by the evolution of free energy profiles of bistable, metastable and super-critical DNA segments. The main contribution to this point’s position comes from the super-critical profile, as becomes evident when comparing energy values shown in Figure 3a,c,e. The observed point movement is related to gradual transformation of bi- and metastable profiles tending toward super-critical state. With increase in kinetic coefficient *L*_χ_ value and corresponding acceleration of structural parameter degradation, the transition process of non-critical profiles to critical one speeds up. When parameter χ reaches critical value χ*_c_*, the metastable DNA segment transitions to super-critical state, accompanied by abrupt change in *Y*’_min_.

The nonlinear nature of the *Y*’_min_(*t*) curve growth indicates periods of relative system stability. Singular behavior correspond to stability loss moments and transitions to new states, fully consistent with the self-organized criticality concept. The presented dynamics clearly illustrates internal field **H** influence playing a key role in synchronizing transitions between different DNA segments and maintaining consistency of changes at the whole genome level.

**Figure 5:**
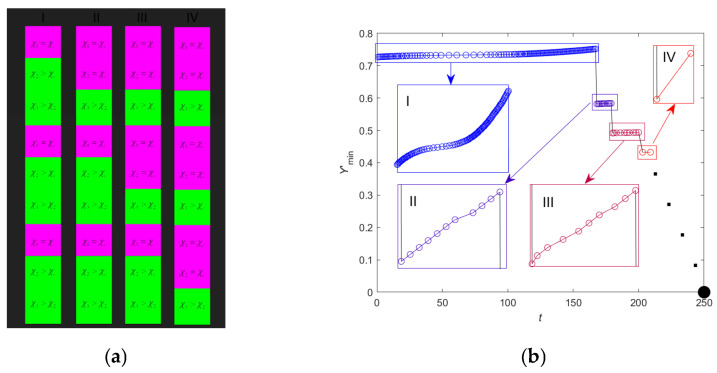
DNA criticality cascade with open states. (**a**) Schematic representation of nine DNA fragments and their stages of its evolution corresponding to the transition of the system to a new state as a result of avalanche formation of open states/gene expression. (**b**) Dependence of *Y*’_min_ on time *t.* In (**a**), I–IV represent sequential stages of the DNA criticality cascade, corresponding to transitions between critical states (proliferation, differentiation, senescence, apoptosis) driven by changes in the structural parameter χ and cooperative open-state behavior.

A question arises about system behavior with an increasing number of interacting segments. Taking gene size as a basis, the total number of segments comprising the genome reaches 22035. However, for this study’s purposes we limit consideration to a system of nine DNA segments, three for each possible state (see Figure 5a). Figure 5b presents *the criticality cascade* phenomenon—sequences of phase transitions DNA undergoes during the cell life cycle. The criticality cascade represents a chain of interconnected transitions between different critical DNA states, each corresponding to a specific cellular state such as proliferation, differentiation, G_0_ phase, aging or apoptosis. These transitions are caused by structural parameter χ changes and cooperative behavior of DNA open states.

In a cellular context, the metastable state corresponds to the proliferation process when the system demonstrates high sensitivity to external signals, ensuring intensive growth and cell division. Transition to a bistable state allows cell stabilization in a new phenotype during differentiation, accompanied by activation of specific genes. The critical state (χ = 1) corresponds to irreversible changes including DNA damage accumulation, ultimately leading to division cessation or programmed cell death initiation.

The phenomenon of criticality cascade offers an explanation for the well-known Hayflick limit [42]—limiting the number of somatic cell divisions (approximately 50–70 times). The molecular mechanism underlying this limit is associated with telomere shortening to a critical length and subsequent activation of the p53/p21 genes, which leads the cell first to a state of senescence and then to apoptosis [43]. In terms of the model under consideration, telomere shortening can be interpreted as an accumulation of open states causing degradation of the structural parameter χ. This, in turn, leads to a gradual loss of the system’s ability to return to the initial state after critical transitions (emergence of new supercritical DNA sites). As a result, the cell loses the ability to proliferate and successively passes into the states of senescence and apoptosis.

Our model suggests that uncontrolled cancerous proliferation corresponds to the disruption of normal criticality cascade flow. The system “gets stuck” in metastable and bistable states, correspondent to proliferation processes without subsequent differentiation. The number of super-critical DNA segments appears insufficient to maintain a normal cell fate change scenario. These observations fully agree with experimental data showing that in cancer cells the critical point often remains in an inactive (OFF) state, preventing global gene expression changes characteristic of normal cellular transitions. Cancer’s disrupted criticality cascade parallels recent findings [19], where EGF stimulation failed to activate the Maxwell’s demon-like function of CP genes. This aligns with our observation that cancer cells ‘stall’ in metastable states due to χ degradation. Zimatore et al. [41] further linked such stalls to impaired chromatin transitions, suggesting that therapeutic strategies targeting both χ stability (mechanical) and entropy flows (thermodynamic) could restore normal criticality.

The criticality cascade represents a fundamental mechanism reflecting the dynamic nature of the genome where consecutive transitions between critical states determine cell fate changes. This phenomenon establishes a direct connection between DNA mechanobiological properties characterized by parameter χ and epigenetic regulation processes, explaining both stable and irreversible cellular changes. Disruptions of normal criticality cascade ending up in system fixation in a given state may underlie various pathological processes including oncological diseases and premature aging. Thus, the criticality cascade serves as a universal physical-biological mechanism integrating molecular and systemic aspects of genome regulation throughout the entire cell life cycle and ensuring consistency of changes at different biological organization levels—from DNA conformational rearrangements to global cellular phenotype transformations. It is worth noting how the need for a continuous adaptation to an unpredictable changing environment is the signature of any homeostatic biological process at any organization layer from macromolecules to ecosystems [44,45,46]. This points to a universal principle of the physics of life, that in the analyzed case was exploited at the genome expression level.

### 2.7. Experimental Validation of Model Representations

Experimental validation of model representations constitutes a crucial stage in the development and verification of theoretical models, particularly for complex biological systems. In this section, we examine potential approaches for experimentally verifying the proposed mechanobiological model that describes DNA’s self-organized criticality and its influence on gene expression regulation.

One primary source of experimental data for model validation comes from studies [10,11,12,13,14,15], which demonstrated the universality of self-organized criticality mechanisms in whole-genome gene expression regulation. These investigations employed DNA microarrays to record time-series gene expression levels across various cellular systems including embryonic, immune and cancerous cells. The researchers revealed that gene expression is regulated through coordinated changes in the genome’s critical states, describable as attractor sets in phase space. These findings can be directly compared with predictions from our model, which similarly describes collective behavior of DNA open-state ensembles and their impact on global gene expression changes.

Specifically, given a known gene expression vector ε*^i^*, we can establish a free energy profile dependent on *Y*’. Assuming a linear relationship between gene expression levels and average open-state size (ε*^i^* = *kY*’*^i^*), we derive the connection between their statistical sums:

(3) $$Z\left(Y\prime^{i}\right)=Z\left(\varepsilon^{i}\right)\left|\frac{d\varepsilon^{i}}{dY\prime^{i}}\right|=kZ\left(\varepsilon^{i}\right),$$

where *k* represents transcription efficiency (mRNA production rate per given average DNA open state). This relation (3) follows from the fundamental theorem of random variable transformations [47]. Consequently, the open-state ensemble-dependent free energy becomes:

(4) $$F\left(Y\prime^{i},f\prime^{i},\chi^{i}\right)=-\Theta \ln\left(Z\left(Y\prime^{i}\right)\right)\approx -\Theta \ln\left(kZ\left(\varepsilon^{i}\right)\right)\approx -\Theta \ln\left(k\hat{f}\left(\varepsilon^{i}\right)\right).$$

For more detailed model validation—particularly given its fundamentally mechanical nature—we propose experiments investigating DNA’s mechanical and chemical properties under conditions simulating cellular environments. Mechanical testing would require nuclear extraction from cells. Such experiments could involve shear flow exposure while tracking nuclear center-of-mass displacement vectors, which may correlate with parameter *Y*’. For instance, quantitative phase microscopy could measure nuclear deformations under fluid flow-induced stress [48,49,50]. Alternative approaches might employ specialized rheometers enabling precise control and measurement of biological sample mechanical properties [51].

To simulate cell fate changes experimentally, chemical agents could be introduced to induce transitions between cellular states like proliferation, differentiation or apoptosis. Differentiation-inducing compounds could be administered while monitoring concomitant changes in gene expression and DNA mechanical properties. Such experiments would test whether structural parameter χ variations—describing collective DNA open-state behavior—correlate with observed cell fate alterations.

Furthermore, potential experimental corroboration could involve magnetic tweezers to measure force-induced open states and fluorescence techniques for dynamic monitoring as well as atomic force microscopy (AFM) could directly observe DNA open states and their dynamics, complementing our simulations. AFM could measure local DNA deformations caused by open-state formation and compare them with model predictions. Fluorescence microscopy might visualize DNA-protein interactions (e.g., with transcription factors or polymerases), elucidating mechanisms underlying open-state collective behavior.

Another crucial validation aspect involves studying epigenetic factors’ influence on DNA open-state dynamics. Chromatin modifications like histone methylation or acetylation might affect structural parameter χ and consequently open-state ensemble behavior. Techniques like ChIP-seq (chromatin immunoprecipitation sequencing) could map protein-DNA binding sites and epigenetic modification levels. Comparing such data with model predictions would assess how accurately the model describes epigenetic influences on gene expression regulation.

Thus, experimental validation of our mechanobiological DNA model with open states would involve comparing theoretical predictions with existing experimental data while developing new experiments to test key model aspects. This approach would not only verify model adequacy but also deepen our understanding of DNA self-organized criticality mechanisms and their role in cellular process regulation.

## 3. Discussion

The present research examined the mechanisms of self-organized whole-genome gene expression regulation using a mechanobiological DNA model with open states. As noted in the Introduction, a key challenge at the biology–physics interface remains the absence of consensus models connecting system energy characteristics with observed gene expression patterns. To address this, we proposed a nonlinear model accounting for collective behavior of DNA open-state ensembles, based on statistical physics and non-equilibrium thermodynamics frameworks. Our approach combined theoretical modeling with numerical methods including parameter sensitivity analysis and structural parameter χ dynamics investigation.

The results demonstrate that our model successfully describes three characteristic DNA states—bistable, metastable and critical—each corresponding to distinct genome regulation regimes. These states evolve and mutually influence one another through structural parameter χ. These findings align well with those of previous studies [10,11,12,13,14,15], where coordinated whole-genome gene expression regulation—mediated by cyclic expression flows between gene groups in bistable, metastable and critical states—was experimentally shown to drive cell fate changes in embryonic, immune and cancerous cell populations. In addition, recent work [52,53] has linked global DNA properties to thermodynamic functions, supporting our model’s approach. Particularly significant is the identified criticality cascade effect, providing physical justification for experimentally observed avalanche-like gene expression changes. The proposed model framework applies not only to normal cellular processes but also to pathological conditions like cancer, where normal critical transition sequences become disrupted.

Analysis of our results leads to the conclusion that structural parameter χ serves as a key regulator of cell fate, governing transitions between different DNA states. A study limitation involves simplified genome representation as a limited number of interacting segments, though this could be addressed in future work by increasing segment numbers and incorporating chemical stimuli. Promising research directions include experimental model validation across cell types; investigating epigenetic factors’ influence on parameter χ; and developing methods for directed cell fate control through modulation of DNA critical states.

## 4. Materials and Methods

### Mechanobiological Model of DNA with Open States

In [25], the authors propose a mechanobiological model of DNA, explicitly accounting for the emergence and interaction of open states in a representative DNA segment. This model allows for the description of DNA mechanical behavior associated with its biological functions. The size of the representative segment can range from the length of a single gene to kilo- or even megabase pairs, making the model applicable for describing the behavior of the entire genome.

The players entering the mathematical formulation of the model are: an internal field **H**; a macroscopic parameter **Y**, describing the ensemble of open states in a representative DNA segment; the dependence of the thermodynamic potential (Helmholtz free energy) Ψ on the macroscopic parameter **Y**; and a structural parameter χ, which determines the type of collective behavior of open-state ensembles. The field **H** is represented as:

(5) $$H=f+J\left(Y-y\right),$$

The relation (5) captures how DNA’s physical state is not just about one gene but involves teamwork across many regions. For example, when a cell needs to activate genes for division, the opening of one DNA segment can trigger a cascade that makes other genes accessible, coordinating the cell’s behavior.

In Equation (5), the first term on the right-hand side of relation describes the external force **f** acting on a nucleotide pair, and the second term represents the long-range influence of all base pairs on the representative DNA segment, caused by stacking interactions, dipole moments, and covalent bonds within the sugar-phosphate backbone. The parameter *J* is the coupling parameter between a specific nucleotide pair and all other pairs in the representative DNA segment. The macroscopic parameter **Y** is the result of averaging the displacement vectors y over the statistical ensemble of states. The difference **Y** − **y** describes the influence of nucleotides on base displacement in a specific pair. If all pairs in the DNA segment are either open or closed, this term equals zero.

The macroscopic parameter **Y** is an internal thermodynamic variable of the ensemble of DNA open states in the space of all possible base displacements relative to their equilibrium positions Ω. It reflects how ready a DNA segment is for gene expression. A high **Y** means the DNA is more open, making it easier for proteins like RNA polymerase to access genes and produce mRNA. This is critical for processes like cell differentiation, where specific genes need to be turned on at the right time. The parameter **Y** is defined as the integral value of base displacements in the representative DNA segment, characterized by vectors **y**, through the self-consistency equation (see [25] for more details).

(6) $$Y=N\int_{\Omega }yZ^{-1}\exp\left(\frac{H\cdot y-D\alpha^{2}y\cdot y}{\Theta }\right)d\Omega ,$$

(7) $$Z=\int_{\Omega }\exp\left(\frac{H\cdot y-D\alpha^{2}y\cdot y}{\Theta }\right)d\Omega ,$$

In the above equations, *N* is the number of interacting open states, *Z* is the partition function, and Θ is the “effective temperature factor” characterizing the current average energy of the system at the specific internal field **H**. Parameter *D* is the dissociation energy of a nucleotide pair, and α is the spatial scale (width) of the potential describing the energy change in a nucleotide pair due to the formation of an open state (the second term in the numerator). Introducing the following dimensionless parameters:

(8) $$Y\prime =\sqrt{\frac{D\alpha^{2}+J}{\Theta }}Y,\;\;\;\;\;\;y\prime =\sqrt{\frac{D\alpha^{2}+J}{\Theta }}y,\;\;\;\;\;\;f\prime =\frac{f}{\sqrt{\Theta \left(D\alpha^{2}+J\right)}},$$

(9) $$\chi =\frac{D\alpha^{2}+J}{J},$$

yield a dimensionless structural parameter χ, which serves as a “thermalization parameter” of DNA. As shown in [25], χ reflects “statistical self-similarity” in the behavior of open states as the “defects” corresponding to different critical regimes associated with qualitatively distinct types of collective modes (attractor states). These modes correspond to the Waddington epigenetic landscape of the mesoscopic DNA state [24]. The structural parameter χ is key to understanding how DNA’s physical state influences gene expression. For instance, a low χ might keep DNA tightly closed, limiting gene activity (e.g., in a dormant cell), while a high χ could make DNA more flexible, enabling rapid gene activation during cell division.

The free energy is assumed to depend on the formation and interaction of open states, the temperature of the DNA segment, and the structural parameter. The dependence of the thermodynamic potential (the Helmholtz free energy) Ψ on the macroscopic parameter **Y** is given by:

(10) $$\begin{array}{l} \Psi =\left(\frac{1}{\chi }-1\right)\frac{Y\prime \cdot Y\prime }{2}-u_{0}\;\ln\left(u_{1}+u_{2}\left|Y\prime \right|+u_{3}Y\prime \cdot Y\prime \right)\\ -u_{4}\left|Y\prime \right|+f\prime \cdot Y\prime -\frac{1}{2}A\prime {\left(\nabla Y\prime \right)}^{2} \end{array},$$

where *A*’ represents the elastic constant of the covalent bond in the sugar-phosphate backbone and is proportional to the square of the characteristic nonlocality scale, and *u*_0–4_ are coefficients approximating the solution of the self-consistence equation (see [20] for more details), with their values listed in Table A1 (Appendix B). The free energy profile explains why DNA can switch between states like bistable (to stable options, like ON/OFF), metastable (one stable and one unstable), or critical (highly sensitive to changes). These states correspond to different cellular behaviors, such as a stem cell choosing to differentiate (bistable) or a cancer cell undergoing rapid, uncontrolled changes (critical).

**Figure 6:**
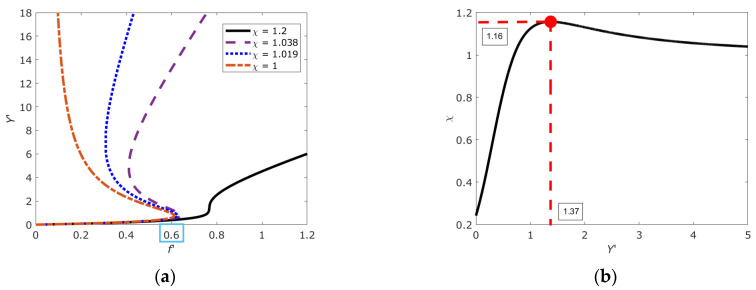
(**a**) Reactions of a representative section of DNA at different values of the structural parameter. *x*-axis: *f*’ (force), *y*-axis: *Y*’ (an internal thermodynamic variable of the ensemble of DNA open states). Different lines correspond to distinct DNA regimes: the dashed line indicates the sub-critical (bistable) state, the dotted line corresponds to the near-critical (metastable) state, and the dash–dotted line represents the super-critical state. (**b**) Dependence of the structural parameter χ on the macroscopic vector of DNA open states *Y*′.

Following linear thermodynamics, the energy dissipation function and the complete system of evolutionary (constitutive) equations for the representative DNA segment with open states can be derived (See Appendix A and Appendix B). The formalism reported in Appendix B generates (consistently with [39]) two critical values of the structural parameter, relative to which DNA demonstrates qualitatively different behavior (χ*_c_*; χ*_t_*) equal to (1; 1.16), respectively (see Figure 6; the critical value of χ*_t_* was defined as the maximum value of the structural parameter at which the second derivative of the free energy potential turns to zero, see Figure 6b).

The structural parameter χ acts as an “effective temperature” of the system. “Effective temperature” refers to the new conditions for the transition (“thermalization”) of an out-of-equilibrium system between different structural states, depending on the magnitude of a certain structural parameter. Thus, DNA dynamics are determined by the collective behavior of open-state ensembles, mirrored by the structural parameter χ.

**Figure 7:**
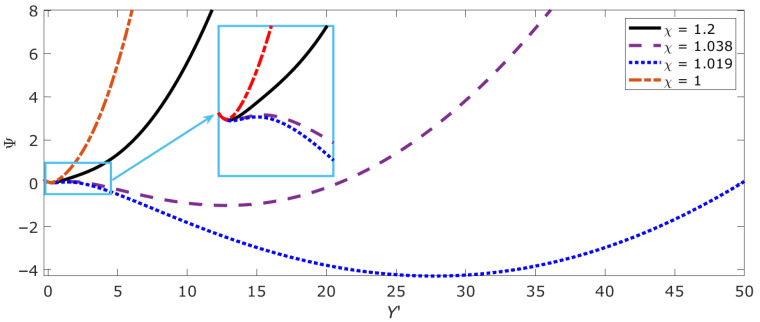
Profiles of the thermodynamic potential of a representative section of DNA with open states at different values of the structural parameter.

Figure 7 shows the free energy profiles Ψ(*Y*’) for different values of the structural parameter presented in Figure 6a and corresponding to transitions between three characteristic nonlinear regimes associated with a change in the symmetry of the system due to the accumulation and interaction of numerous open states. As can be seen from Figure 7 (transition between the dashed and dotted lines), the tendency of χ to unity corresponds to a scenario in which the DNA site can completely melt (this corresponds to a large value of *Y*’). This result allows us to conclude that the parameter χ defines new conditions of “thermalization” of the system, which do not depend on the usual thermalization conditions, i.e., temperature, but on its internal configuration (in synthesis: the higher the number of open states, the higher the ‘temperature’).

As noted above, the epigenetic landscape (in its basic form of open/close to transcription DNA patches) is a proxy of the energy profile of DNA molecule. Relation (10) describes the epigenetic landscape in terms of the free energy of DNA with open states. The parameter χ, along with (configurational) temperature, plays the role of a parameter that determines the structure of the landscape. The transition between the three possible scenarios presented in Figure 6 and Figure 7 correlates with the results reported in [11,12,54] and provides an explanation for the mechanism generating different gene expression states. In particular, with χ varying between 1.1 and 1, the free energy profile Ψ shows how the epigenetic landscape gradually changes from a bistable (sub-critical) free energy profile, passing through a (near-critical) profile with metastable and stable regions, to a (super-) critical free energy profile. In Figure 7, the sub-critical dependence with two stable minima corresponds to the dashed line, the near-critical curve with one unstable and one stable minimum corresponds to the dashed line, and the super-critical state corresponds to the dashed–dotted line.

## Data Availability

The datasets analyzed during the current study are available from the corresponding author on request.

## Appendix A

The representation of the thermodynamic potential Ψ can be expressed by means of a fourth-order decomposition analogous to the well-known Ginzburg–Landau decomposition:

(A1) $$\Psi =-\frac{A}{2}{\left(\nabla Y\prime \right)}^{2}+B\left(1-\frac{\chi }{\chi_{t}}\right)Y\prime^{2}-CY\prime^{3}+D\left(1-\frac{\chi }{\chi_{c}}\right)Y\prime^{4}-f\prime Y\prime .$$

Bifurcation points χ*_t_* and χ*_c_* play a role analogous to characteristic temperatures in Landau’s theory of phase transitions. The gradient term in (A1) describes the effects of nonlocality in the ensemble of open states in the so-called long-wave approximation; *B*, *C*, *D* are phenomenological coefficients. Then the kinetic Equation (21)_1_ is transformed to the form:

(A2) $$\dot{Y}\prime =-L_{Y}\left(-A\Delta Y\prime +2B\left(1-\frac{\chi }{\chi_{t}}\right)Y\prime -3CY\prime^{2}+4D\left(1-\frac{\chi }{\chi_{c}}\right)Y\prime^{3}-f\prime \right).$$

As illustrated in Figure 7, transitions through the bifurcation points χ*_t_* and χ*_c_* result in a pronounced alteration in the shape of the free energy potential Ψ. Consequently, this leads to the emergence of specific (collective) modes in the system. The impact of the mentioned transitions on the evolution of the ensemble of open states is determined by the type of bifurcation—group properties of the equation of motion (A2) for different regions with χ (χ > χ*_t_*, χ*_t_* < χ < χ*_c_*, χ < χ*_c_*).

When χ > χ*_t_*, the system is in a state of quasi-equilibrium, and the *Y*’ is found to depend only on the spatial coordinates (d*Y*’/d*t* = 0). The Equation (A2) corresponding to χ > χ*_t_* has a periodic solution (breathers):

(A3) $$Y\prime =Y\prime_{0}\cos\left(z{\left(\frac{A}{\left(2B\left(1-\chi /\chi_{t}\right)\right)}\right)}^{-1/2}\right).$$

It describes the weak oscillations of the open states associated with the anisotropy induced by the external force *f’*. The period depends on the structural parameter χ and increases as one approaches the bifurcation point χ*_t_*, which corresponds to the scale divergence, the period ~ ln(χ − χ*_t_*).

At χ → χ*_t_*, the coefficient on the linear term 2*B*(1 − χ/χ*_t_*) tends to zero. This leads to a qualitative change in the form of the free energy potential. The solution of elliptic equation passes into a separatrix that connects the equilibrium points of the potential, and the solution is transformed into a self-similar solution *Y*’(ζ), where ζ = *z* − *Vt* is the self-similar variable and *V* is the velocity of the wave front. The self-similar solution (autosoliton) is a localized perturbation that preserves its shape and propagates with a constant velocity in the non-conservative system due to the balance between nonlinearity and the processes of energy storage and dissipation. Then, the kinetic Equation (A2) is transformed into a stationary equation depending on the variable ζ:

(A4) $$-V\frac{dY\prime }{d\zeta }=-L_{Y}\left(Y\prime \left(Y\prime -Y\prime_{t}\right)\left(Y\prime -Y\prime_{c}\right)-f\prime \right)+L_{Y}A\frac{d^{2}Y\prime }{d\zeta^{2}},$$

where the parameters *Y*’*_t_* and *Y*’*_c_* are given by relations

(A5) $$Y\prime_{t}Y\prime_{c}=\frac{B\left(1-\frac{\chi }{\chi_{t}}\right)}{2D\left(1-\frac{\chi }{\chi_{c}}\right)},$$

(A6) $$Y\prime_{t}+Y\prime_{c}=\frac{3C}{4D\left(1-\frac{\chi }{\chi_{c}}\right)}.$$

The form of the self-similar solution of Equation (A4) is assumed to be as follows:

(A7) $$Y\prime \left(\zeta \right)=\frac{1}{2}Y\prime_{c}\left(1-\tanh\left(2\zeta l^{-1}\right)\right),$$

where *l* is the width of the front and *Y*’*_c_* is the amplitude, defined as

(A8) $$l=\frac{4}{Y\prime_{c}}\sqrt{2L_{Y}A},$$

(A9) $$V=\sqrt{\frac{L_{Y}A}{2}}\left(Y\prime_{c}-2Y\prime_{t}\right).$$

The autosoliton occurs when the system transitions from a stationary state to a dynamic state where time evolution is taken into account. This is due to the fact that Equation (A4) describes equilibrium configurations, but at χ → χ*_t_* the system becomes unstable, and to describe the transition to a new state it is necessary to take into account the kinetics given by Equation (A4).

## Appendix B

Referring to the relations (5)–(10) reported in the text it is possible to derive the energy dissipation function and the constitutive equations for a DNA segment with open states

(A10) $$\dot{Y}\cdot \left(f-\frac{\partial F}{\partial Y}-A\Delta Y\right)-\dot{\chi }\frac{\partial F}{\partial \chi }-\dot{c}\frac{\partial F}{\partial c}\geq 0,$$

(A11) $$\left\{\begin{array}{l} \dot{Y}\prime =-L_{Y}\left(\left(\chi^{-1}-1\right)\cdot Y\prime -\frac{P_{3}\left(Y\prime \right)}{M_{4}\left(Y\prime \right)}-u_{4}+f\prime -A\prime \Delta Y\prime \right)\\ \dot{\chi }=-\left(L_{\chi }+\frac{{L_{c\chi }}^{2}}{L_{c}}\right)\frac{H\left(\chi -1\right)Y\prime \cdot Y\prime }{2\chi^{2}}-\frac{L_{c\chi }}{L_{c}}\dot{c}\\ Y\prime \left(z,0\right)=Y\prime_{0},\;\chi \left(z,0\right)=\chi_{0}\\ {\left.{\left.\frac{\partial Y\prime }{\partial z}\right|}_{z\to 1}=y_{1},{\left.\;\frac{\partial Y\prime }{\partial z}\right|}_{z\to G}=y_{G},\frac{\partial \chi }{\partial z}\right|}_{z\to 1}=\chi_{1},\;{\left.\frac{\partial \chi }{\partial z}\right|}_{z\to G}=\chi_{G} \end{array}\right.,$$

where *c* is the level of an internal or external chemical signal (e.g., concentration of a substance/protein) influencing the formation of open or closed states (e.g., a methyl group or RNA polymerase, respectively), *L***_Y_**, *L_χ_*, *L_cχ_* are positive kinetic coefficients, *P*_3_/*M*_4_ − *u*_4_ is the Padé coefficient approximating the dimensionless internal field, *z* and *G* are the number and quantity of representative DNA segments, and *H*(∙) is the Heaviside function. Boundary conditions of the second kind are used, which can be interpreted as an external field generated by the “boundary” DNA segment.

The equilibrium responses of DNA in the coordinates of the modulus of the macroscopic vector *Y*’ = |**Y**’| as a function of the structural parameter χ are shown in Figure 6a. The dependencies illustrate the change in the thermodynamic variable *Y*’ under the applied force *f*’ = |**f**’| without accounting for nonlocality effects, at various values of χ corresponding to different DNA responses. The polynomials *P*_3_ and *M*_4_ have the following forms:

(A12) $$P_{3}\left(Y\prime \right)=p_{3}Y\prime^{3}+p_{2}Y\prime^{2}+p_{1}Y\prime +p_{0},$$

(A13) $$M_{4}\left(Y\prime \right)=m_{4}Y\prime^{4}+m_{3}Y\prime^{3}+m_{2}Y\prime^{2}+m_{1}Y\prime +m_{0}$$

with coefficient values provided in Table A1.

**Table A1:** Values of the coefficients of the Pade approximant.

I	0	1	2	3	4
*UI*	0.6	1.1	0.6	1	0.04
*PI*	0.07	23.14	18.77	0.8	-
*MI*	8.2	25.7	18.49	19.5	−0.06
